# Large-Scale Evaluation and Liver Disease Risk Prediction in Finland’s National Electronic Health Record System: Feasibility Study Using Real-World Data

**DOI:** 10.2196/62978

**Published:** 2025-04-02

**Authors:** Viljami Männikkö, Janne Tommola, Emmi Tikkanen, Olli-Pekka Hätinen, Fredrik Åberg

**Affiliations:** 1 Atostek Oy Tampere Finland; 2 Faculty of Medicine and Health Technology Tampere University (TUNI) Tampere Finland; 3 Pfizer Oy Helsinki Finland; 4 Transplantation and Liver Surgery Unit Helsinki University Hospital and University of Helsinki Helsinki Finland

**Keywords:** Kanta archive, national patient data repository, real world data, risk prediction, chronic liver disease, mortality, risk detection, alcoholic liver, prediction, obesity, overweight, electronic health record, wearables, smartwatch

## Abstract

**Background:**

Globally, the incidence and mortality of chronic liver disease are escalating. Early detection of liver disease remains a challenge, often occurring at symptomatic stages when preventative measures are less effective. The Chronic Liver Disease score (CLivD) is a predictive risk model developed using Finnish health care data, aiming to forecast an individual’s risk of developing chronic liver disease in subsequent years. The Kanta Service is a national electronic health record system in Finland that stores comprehensive health care data including patient medical histories, prescriptions, and laboratory results, to facilitate health care delivery and research.

**Objective:**

This study aimed to evaluate the feasibility of implementing an automatic CLivD score with the current Kanta platform and identify and suggest improvements for Kanta that would enable accurate automatic risk detection.

**Methods:**

In this study, a real-world data repository (Kanta) was used as a data source for “The ClivD score” risk calculation model. Our dataset consisted of 96,200 individuals’ whole medical history from Kanta. For real-world data use, we designed processes to handle missing input in the calculation process.

**Results:**

We found that Kanta currently lacks many CLivD risk model input parameters in the structured format required to calculate precise risk scores. However, the risk scores can be improved by using the unstructured text in patient reports and by approximating variables by using other health data–like diagnosis information. Using structured data, we were able to identify only 33 out of 51,275 individuals in the “low risk” category and 308 out of 51,275 individuals (<1%) in the “moderate risk” category. By adding diagnosis information approximation and free text use, we were able to identify 18,895 out of 51,275 (37%) individuals in the “low risk” category and 2125 out of 51,275 (4%) individuals in the “moderate risk” category. In both cases, we were not able to identify any individuals in the “high-risk” category because of the missing waist-hip ratio measurement. We evaluated 3 scenarios to improve the coverage of waist-hip ratio data in Kanta and these yielded the most substantial improvement in prediction accuracy.

**Conclusions:**

We conclude that the current structured Kanta data is not enough for precise risk calculation for CLivD or other diseases where obesity, smoking, and alcohol use are important risk factors. Our simulations show up to 14% improvement in risk detection when adding support for missing input variables. Kanta shows the potential for implementing nationwide automated risk detection models that could result in improved disease prevention and public health.

## Introduction

### Background

Even though health care risk models have been developed for a very long time and have been implemented to be available for individuals, health care still lacks automated health-risk analysis because of limited real-world data (RWD). The burden of liver disease increases yearly in Finland because the Finnish population age average grows, and obesity and overweight are more common problems in the Finnish population [1]. On average, there are 1000 deaths caused by alcoholic liver disease every year [2]. For the early detection of individuals from the general population at high risk for future severe liver disease, the CLivD (Chronic Liver Disease score) score was developed. It can be used to predict severe liver disease incidence in 10 years. The model itself was developed by linking data from Finnish population–based health examination surveys “FINRISK” and “Health 2000” with Finnish health care registries. The model has been validated using data from the United Kingdom, Denmark, the United States, and China [1,3-5].

### Risk Model Application to RWD

**Figure 1:**
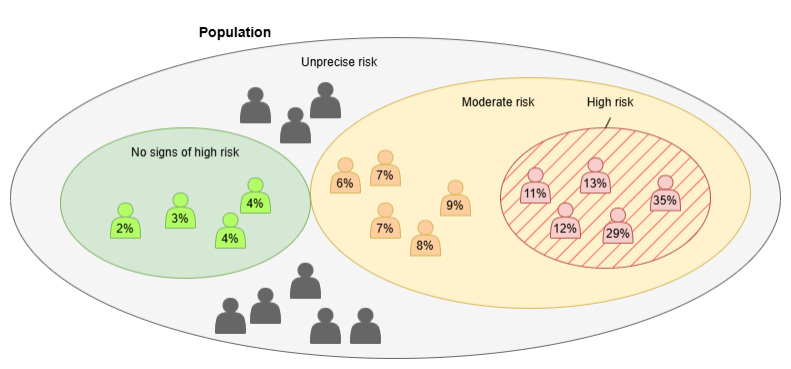
Case example of population risk categorization based on a personal risk value.

The missing input data pose a challenge for the large-scale implementation of established risk models in real life [1]. To be able to calculate the exact risk value in many cases, we would not only require additional RWD sources but also changes in health care professionals’ practices to ensure they regularly measure the correct parameters from patients. One problem is the negation of information, particularly in cases where data on behaviors, such as a person not smoking or not consuming alcohol, is missing. This leads to a problem where we cannot determine whether a person, for example, is a nonsmoker if there is no record of it. Despite the challenges, health care RWD enables early identification of individuals at high risk by analyzing their current characteristics, which is crucial for health care professionals in planning treatments. The exact risk value would be easy for everyday people to understand, but for health care professionals in preventive work and treatment planning, it is also important to detect potential high-risk cases as early as possible and identify if the person is a high-risk patient. For this kind of usage, we introduce a process that helps to detect high-risk and potential high-risk individuals. Figure 1 describes that kind of division at the population level.

### The Finnish National Electronic Health Record System

Kanta Services is the Finnish National Electronic health record system where data are recorded from almost all Finnish health care providers including the public and private sectors and primary and special care [6]. Kanta Services entered production in Finland in 2010 and it has been used for more than 10 years in Finland overall [6]. However, Kanta has been developed in stages, resulting in different data types having different levels of availability. Kanta Services in general consists of four different parts: (1) the social health care part, (2) the prescription center, (3) the patient data repository (PDR), and (4) personal health records [7]. In this study, we only use data from the Kanta PDR.

In Finland, the 2019 Act on Secondary Law made it possible to use health care data for research purposes [8]. Before that, the data were available only for their primary use, which is patient care. Kanta is an exceptional RWD source because it covers almost the whole Finnish population and has recordings from more than 90% of all Finnish health care service providers. [6] This makes it possible to analyze previously developed health care risk models and develop new risk models by using large data sources that represent the entire national population. Since data are continuously generated in Finnish day-to-day health care and all Finnish health care systems are integrated into Kanta, developed and tested risk models can also be applied in actual patient care. Popular well-being service providers have implemented automated health risk analysis based on data collected from the person regularly or data obtained from wearable devices like smartwatches [9]. These types of data still lack validation from health care professionals. Validation is a key element for incorporating automated risk analysis results in everyday patient care and using them as a diagnostic tool. The Kanta PDR contains only data validated by health care professionals, as only they are authorized to record information in the system [10]. Kanta Services also has a personal health records data repository containing data recorded by the people themselves. However, it is not used in this research as it is still under development. In this study, we aim to analyze data validated by health care professionals [11].

## Methods

### Overview

In this study, we aim to research the CLivD score risk model automation possibilities with Kanta PDR. We use Kanta PDR as the only data source for the risk model to have an overall picture of the data availability status. We consider four different scenarios of data usage possibilities: (1) first, we test the risk model results with the available structured data; (2) next, we aim to use other structured health care information to approximate missing information; (3) after that, we analyze the possibilities of using free text; and (4) finally, we analyze completely missing input variables. For this research, we have 2 main objectives:

1. To evaluate the feasibility of implementing an automatic CLivD score with the current Kanta platform.
2. To identify and suggest improvements for Kanta that would enable accurate automatic risk detection.

### Dataset

In this study, we used the dataset consisting of the medical documents of 192,400 individuals archived in the Kanta PDR between 2014 and mid-2022. Data in Kanta are recorded using the Clinical Document Architecture Release 2 (CDA R2) format, as defined by Health Level 7 (HL7) [12]. CDA R2 documents are XML documents that follow a defined format [13]. In the Finnish health care environment, the local Finnish version of the global HL7 CDA R2 is defined by Health Level 7 Finland, Kela, and the Finnish Institute for Health and Welfare (THL) [12].

The dataset was chosen by Kela from Kanta and the study cohort was randomly selected across the whole Finnish population without any limitations to specific health care providers or locations. The dataset included all documents that were recorded in Kanta PDR after an individual turned 18 years old. Documents were pseudonymized by Findata and delivered in the original CDA R2 XML format to the secure Kapseli environment. Before the analysis was done, the dataset was split randomly into development and validation datasets. Data were evenly divided, with both development and validation datasets containing 96,200 patients. The data were split to accommodate future plans for using machine learning and other methods requiring validation in subsequent projects; however, in this project, only the first half of the data were used. After the data are split, CDA R2 XML documents must be processed so that all relevant data are parsed for analysis. For data processing, a separate data process library and data model were designed. Data parsing consisted of structured laboratory measurements, structured diagnosis data, structured physiological measurements, free-text sections of patients’ ongoing treatment reports, and some basic information about the document and patient. We did not obtain access to death records because they are not recorded in Kanta and would have required separate permission and retrieval from the Digital and Population Data Services agency. A more detailed analysis of the dataset can be found in the study by Männikkö et al [14].

### Risk Model Implementation

The original research on the CLivD score introduced 2 risk calculation models: Model_lab_ and Model_nonlab_. Both models predict the risk of chronic liver disease in people aged 40 years or older. The difference between models is that Model_lab_ also considers the person’s gamma glutamyl transferase (GGT) laboratory test result. Both risk calculators have 4 different exclusion criteria [1]. If a person meets even 1 of the exclusion criteria, there is a possibility that the risk calculator may not function as expected. Exclusion criteria are listed in Textbox 1.

For these risk models and use cases, we introduce a model for categorizing patients to 4 different categories: “low risk,” “moderate risk,” “high risk,” and “not specified risk.” Categorization is based on 2 risk values: “minimum risk” and “maximum risk.” The risk calculation process itself was introduced slightly later. This approach is good from the Kanta development point of view, as future developments and the addition of more information to Kanta will allow risk categorization to be easily applied to the new data. The more data we collect from the person, the more reliable the model becomes, resulting in a significant reduction in the “Not Specified” category. Table 1 presents the cutoff values for each category.

**Chronic liver disease score risk model exclusion criteria.**

**Exclusion criteria**

- Age: above 40 and under 71 years.
- Liver disease diagnosis: *ICD-10* (*International Statistical Classification of Diseases and Related Health Problems, 10th Revision*): K70-K77, C22.0; *ICD-8* (*The International Classification of Diseases, 8th Revision*) and *ICD-9* (*International Classification of Diseases, 9th Revision*): 570-573, 155.0.
- Chronic viral hepatitis diagnosis: *ICD-10*: B18.
- Current alcohol abstainer: previous alcohol use. Can be identified with *ICD-10* codes: F10.20 and other F10.2X.

**Table 1:** Risk categories’ cutoff values for minimum and maximum risk.

Risk	Low risk	Moderate risk	High risk	Not specified risk
Minimum risk	≤5%	>5% and <10%	≥10%	<5%
Maximum risk	<5%	>5%	≥10%	>10%

The CLivD score risk function was developed and validated using cohort studies, where the population is sampled, assessed at a certain time, and then followed for outcomes [1]. Kanta patient data are distributed over time, with measurements conducted at different points in time.

**Figure 2:**
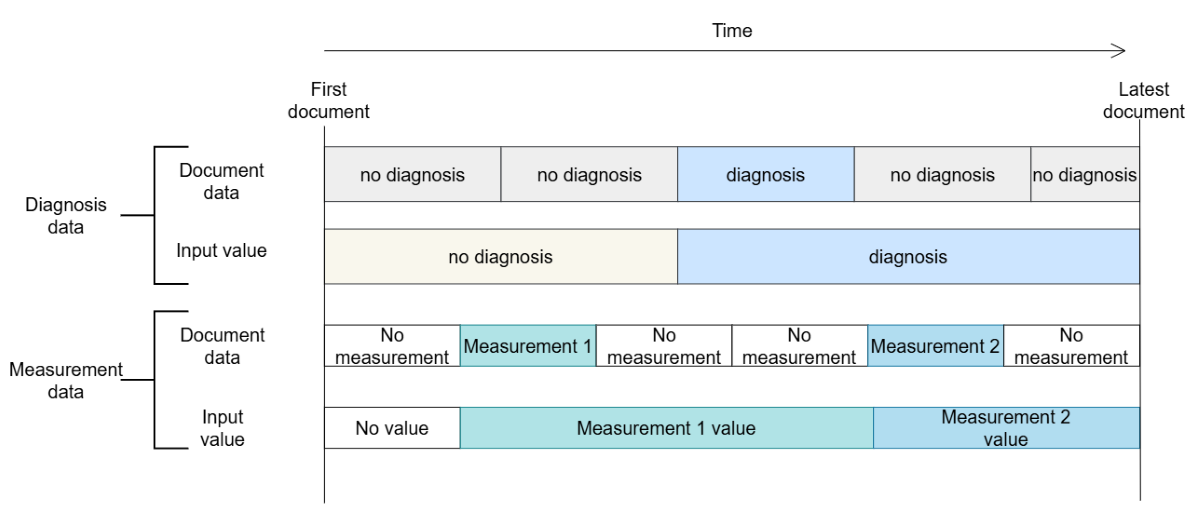
In real-world data, the validity period of input parameters must be defined, as different measurements remain valid for varying durations both before and after the measurement.

We use parameter lifecycles or lifetimes to do this, where we specify the length of time a measurement or diagnosis is valid, both before and after it appears in the medical record. In this work, we used 2 different lifecycles: 1-year lifecycles where measurements are valid for 1 year after measurement, and infinite lifecycles where they stay valid until the next measurement, or until the end of document history. For both lifecycle types, we used an infinite validity time for diagnoses. An example of parameter lifecycles is shown in Figure 2 with a finite (eg, 1 year) lifetime for measurements and an infinite lifetime for diagnoses.

**Figure 3:**
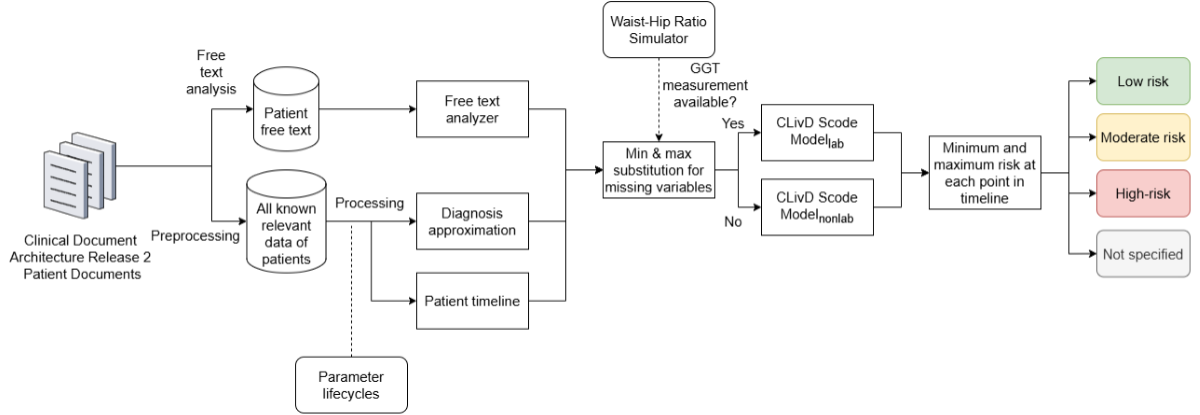
Risk categorization process flow. CLivD: Chronic Liver Disease; GGT: gamma glutamyl transference.

We implemented the CLivD risk function in Kapseli using Python (Python Software Foundation). The implementation flow diagram is described in Figure 3. The timeline is formed by using all known relevant patient data and applying the previously discussed parameter lifecycles to it. Due to missing input variables, we calculate 2 risk values: the minimum and maximum risk. The minimum risk is obtained by substituting the missing variables with their lowest values, while the maximum risk is determined by using their highest value. The complete list of default values can be found in Table 2. Values are used in the case where the input value is not available from an individual’s medical history.

By calculating the minimum risk and maximum risk, we obtain a risk range. A smaller risk range directly implies a more accurate risk value. Missing input parameters increase the risk value range. The goal is to narrow the risk value range toward the actual risk value in the long term. Possible ways to narrow the risk range are, for example, getting the exact missing value from other data sources, asking for information from the patient, or determining the magnitude of the missing information from free-text evaluation.

**Table 2:** Default values for risk calculation when an input parameter is missing.

Input parameter	Default minimum value	Default maximum value
Gender	Always available	Always available
Age (years)	40 (basically, always available)	70 (basically, always available)
GGT^a^	Use non-GGT model	Use non-GGT model
WHR^b^	0.7	1.3
Smoking	False	True
Alcohol usage	0	49
Diabetes	False	True

^a^GGT: gamma glutamyl transferase.

^b^WHR: waist-hip ratio.

**Figure 4:**
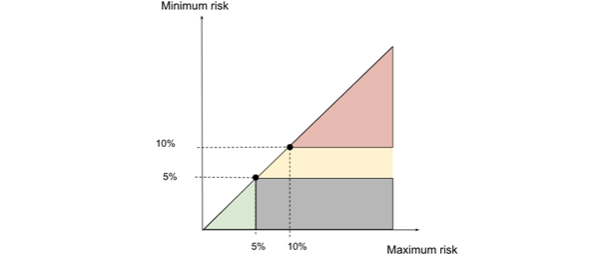
Risk categories in minimum and maximum risk plane.

In Figure 4, an illustration and example goal for the risk value ranges are shown. The line represents an ideal case where all input variables are known, and an exact risk value with equal minimum and maximum risk can be calculated. The lower turquoise triangle represents an area where the maximum risk is below 5%, and the patient can be considered low risk. The upper red triangle represents an area where the minimum risk is above 10%, and the patient is at high risk. The yellow area is the moderate risk area where patients’ risk value is higher than 5% but not high enough to determine the person as a high-risk patient. The gray-colored area represents the “not specified risk” category, where the risk range between the minimum risk and maximum risk is too wide to identify the correct category.

In this study, we target these colored areas as a goal, as an exact risk value is not feasible with the missing data, but a risk range is still helpful for determining the course of action. The risk thresholds can be configured for different use cases. The complete risk calculation formula can be found in Multimedia Appendix 1.

### Ethical Considerations

This study involved pseudonymized health care data produced in health care services in Finland. Data were collected, delivered, and pseudonymized under the legislation on secondary use of health care data in Finland. Finnish authority Findata validates data requests and project plans before granting permission to access data (THL/1851/14.02.00/2021). Findata also has the responsibility to create the data pseudonymization before delivery. Data were processed and analyzed in an audited secure computing environment known as “Kapseli” with restricted access to it. All the results presented in this paper have gone through the Findata anonymization validation process where Findata ensures that results taken out of a secure environment are anonymized. Ethical aspects of the project have been evaluated by Findata during the data request process.

## Results

### Overview

Analysis was made in 4 iterations. At first, we analyzed the structural data and its occurrences. After that, we tried to improve the model with the diagnosis use to determine the magnitude of missing input variables. After that, we used free-text for tobacco and alcohol information, and at last, we simulated waist-hip ratio (WHR) in cases where it would have been available from different data sources.

The study initially included 96,200 participants. After applying the exclusion criteria, only 51,275 eligible individuals remained for the risk model. A total of 44,925 individuals were left out of this analysis because they did not match the original criteria. Almost 50% of the individuals were excluded, with the age-related criteria being the most common reason for exclusion.

### Structural Data

We can analyze the CLivD score risk calculation results using Kanta PDR structured data as a precise input parameter, without approximating the parameter magnitude. In Table 3, we describe the frequency of relevant variables appearing in a patient’s medical history in a structured format. The occurrence of 1 means that the variable has been measured just once during the patient’s medical history according to the Kanta data. Age and gender are registered in every document and are always available. The table shows that 0 measurements are the most common, and more than 5 measurements are a very rare case among risk model input variables.

WHR and alcohol use are not shown in this table as they are not present in a structured format in the Kanta data. Besides age and gender, the only input variables with at least 1% availability are GGT and fasting glucose, with approximately 5% and 13% total occurrence, respectively. As fasting glucose (7 mmol/L) is only used as an alternative to a diabetes diagnosis, it is not a required input variable. We interpret a missing measurement to mean that the patient does not have diabetes, provided the other criteria are not met. BMI and waist circumference are not input variables for the risk model but were investigated as possible alternatives to the missing WHR. Waist circumference measurements are very rare and will not considered further, whereas BMI has moderate availability and will be discussed later.

**Table 3:** Parameter occurrences in the Kanta patient data repository between 2014 and 2022 (N=96,200).

Occurrence during patient history, n	Fasting glucose, n (%)	Height, n (%)	Weight, n (%)	BMI, n (%)	Waist circumference, n (%)	Gamma glutamyl transferase, n (%)	Smoking, n (%)
0	84,521 (87)	76,815 (79)	78,003 (81)	80,651 (83)	95,812 (99)	91,891 (95)	96,193 (99)
1	9141 (10)	10923 (11)	9007 (9)	8482 (9)	349 (<1)	3144 (3)	7 (<1)
2-5	2460 (2)	7752 (8)	7311 (8)	6393 (6)	39 (<1)	1067 (1)	0 (0)
6-10	64 (<1)	608 (1)	1364 (1)	567 (1)	0 (0)	77 (<1)	0 (0)
11-15	8 (<1)	82 (<1)	329 (<1)	85 (<1)	0 (0)	12 (<1)	0 (0)
>15	6 (<1)	20 (<1)	186 (<1)	22 (<1)	0 (0)	9 (<1)	0 (0)

### Diagnosis Use

Because the Kanta PDR is missing WHR data, and alcohol usage data and tobacco usage information are quite rarely found in a structured format, we need to use other health care information found in the Kanta PDR. For alcohol and tobacco usage, there are some *ICD-10* (International Statistical Classification of Diseases and Related Health Problems, 10th Revision) and ICPC-2 (International Classification of Primary Care version 2) diagnosis codes that can be used for risk calculation. With the smoking diagnosis codes, we get the information that an individual smokes. This serves as the exact value for the CLivD score model, as it does not consider the number of cigarettes a person smokes per week.

For alcohol usage, the CLivD score model uses the number of servings as an input. Consequently, by using diagnosis codes, we aim to estimate the magnitude of alcohol usage and narrow the risk range. For alcohol usage, we use diagnosis codes that relate to heavy consumption of alcohol. Based on those diagnosis codes, we can say that the person has consumed alcohol at above-average levels for some time before getting the diagnosis. The largest *ICD-10* group that will be used is F10 “Mental and behavioral disorders due to the use of alcohol” and from the ICPC-2 codes we will use P15 “Chronic alcohol abuse” and P16 “Acute alcohol abuse.” For high-risk alcohol users, we use the “23 alcohol servings per week” approximation for men and the “12 alcohol servings per week” approximation for women [15]. The complete list of diagnosis code mappings to risk model input parameters can be found in Multimedia Appendix 2. Table 4 shows the statistics from diagnosis occurrences in our dataset.

**Table 4:** Alcohol and smoking-related diagnosis occurrences in the Kanta patient data repository between 2014 and 2022 (N=96,200).

Diagnosis code			Display name		Number of diagnoses, n
**Alcohol-related**					
	F10.09	Unspecified alcohol intoxication		2158	
	F10.1	Alcohol abuse		18,141	
	F10.20	Alcohol dependence uncomplicated		3697	
	F10.24	Alcohol dependence with alcohol-induced mood disorder		3251	
	F10.25	Alcohol dependence with alcohol-induced psychotic disorder		3921	
	F10.26	Alcohol dependence with alcohol-induced persisting amnestic disorder		3328	
	F10.29	Alcohol dependence with unspecified alcohol-induced disorder		3268	
	F10.39	Alcohol withdrawal symptoms unspecified		1690	
	P15	Alcohol abuse (chronic)		4494	
	P16	Alcohol abuse (acute)		2417	
**Smoking-related**					
	Z72.0	Tobacco use		1102	
	P17	Tobacco abuse		465	

### Free-Text Analysis

Kanta PDR contains a lot of free text in patients’ ongoing treatment reports that describe the patient’s overall health status and living habits. As a result, the free text can be used to find information concerning alcohol usage habits and tobacco use. Due to the limitations on available resources, such as the lack of a graphics card in a secure “Kapseli” environment, we had limited options for free-text analysis. We were not able to use advanced machine learning models or any generic artificial intelligence for text analysis. Instead of those methods, we used a simple regex-based keyword search and converted found text phrases into usage categories. With this analysis, we aimed to understand how often tobacco- or alcohol-related texts appear in ongoing treatment reports and how it would improve the CLivD score model. For more precise free-text analysis, more advanced tools should be used to gain more reliable results.

Tobacco usage is simpler because we can find texts that indicate whether a person is a smoker or a nonsmoker. There are cases where the texts might state, “person has smoked 10 years ago,” making it difficult to determine whether the person currently smokes without additional context. However, in most cases, we can get quite good results by finding sentences related to smoking status.

Alcohol usage is different than tobacco use. Because alcohol servings are defined in a weekly servings format in the CLivD Score model, we must define alcohol usage categories also for free-text analysis. Based on the analysis, we converted phrases into alcohol usage categories. Category mappings are defined in Multimedia Appendix 3 and are based on the THL alcohol risk table [15]. We noticed that alcohol usage was described in free text using various ways and words. We also noticed that the amount descriptions were, in many cases, abstract, and the conversion to the alcohol usage category was not reliable. However, we managed to find clear and reliable cases of alcohol usage from the free text and managed to use them. For the text finding, we used keywords like “käytä alkohol,” “ei\s*\w*\s*alkohol,” “runsa\w*\s*alcohol,” and “vieroitus|vierotus|päihtymys|putki|katkaisu.” Multimedia Appendix 3 defines the alcohol usage categories used in free-text analysis. The categories are based on THL definitions for alcohol usage risk groups defined as alcohol servings per week. In each category, there is minimum and maximum alcohol usage. In the risk model when calculating the minimum risk, the alcohol usage category minimum value is used and vice versa. A complete list of alcohol usage and tobacco use text analysis keywords and mappings can be found in Multimedia Appendix 4.

### Risk Calculation Results

We apply the risk function to the patient’s timelines and attempt to assign their risks to the colored areas shown in Figure 4. These risk areas would be enough to classify patients into low- and high-risk groups, with the uncolored area representing gray area where no definite conclusions can be made.

**Figure 5:**
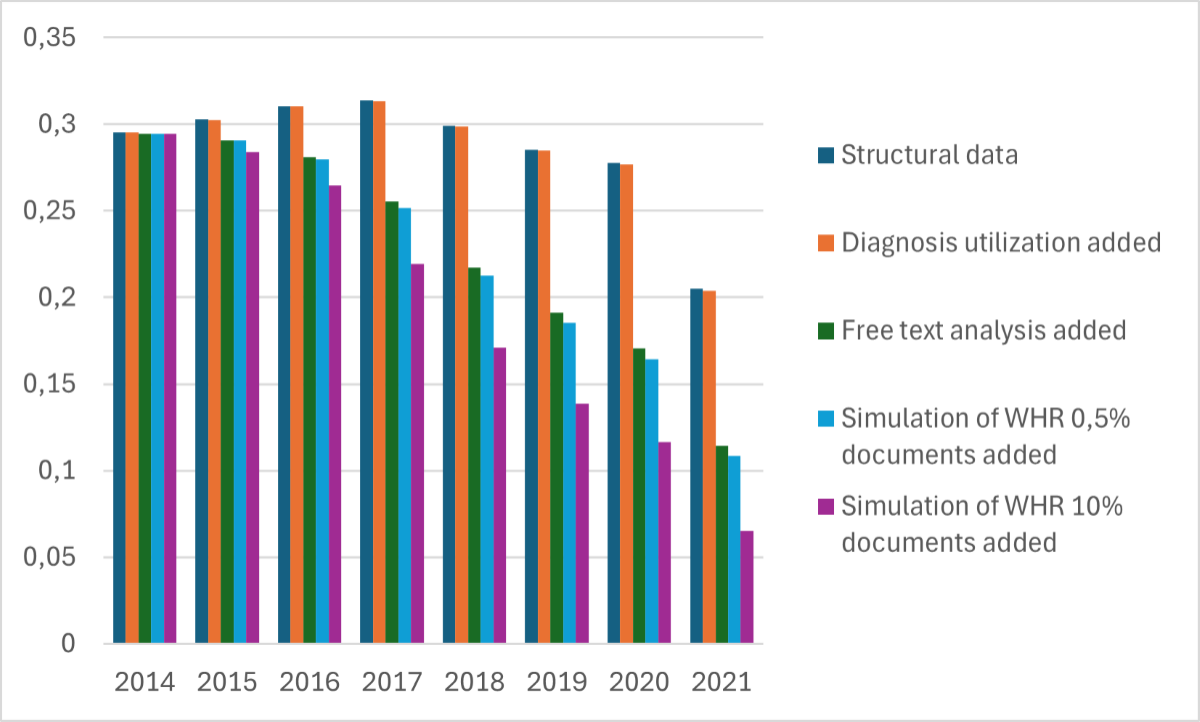
Average risk difference development between 2014 and 2021 with eternal parameter lifetime. WHR: waist-hip ratio.

**Figure 6:**
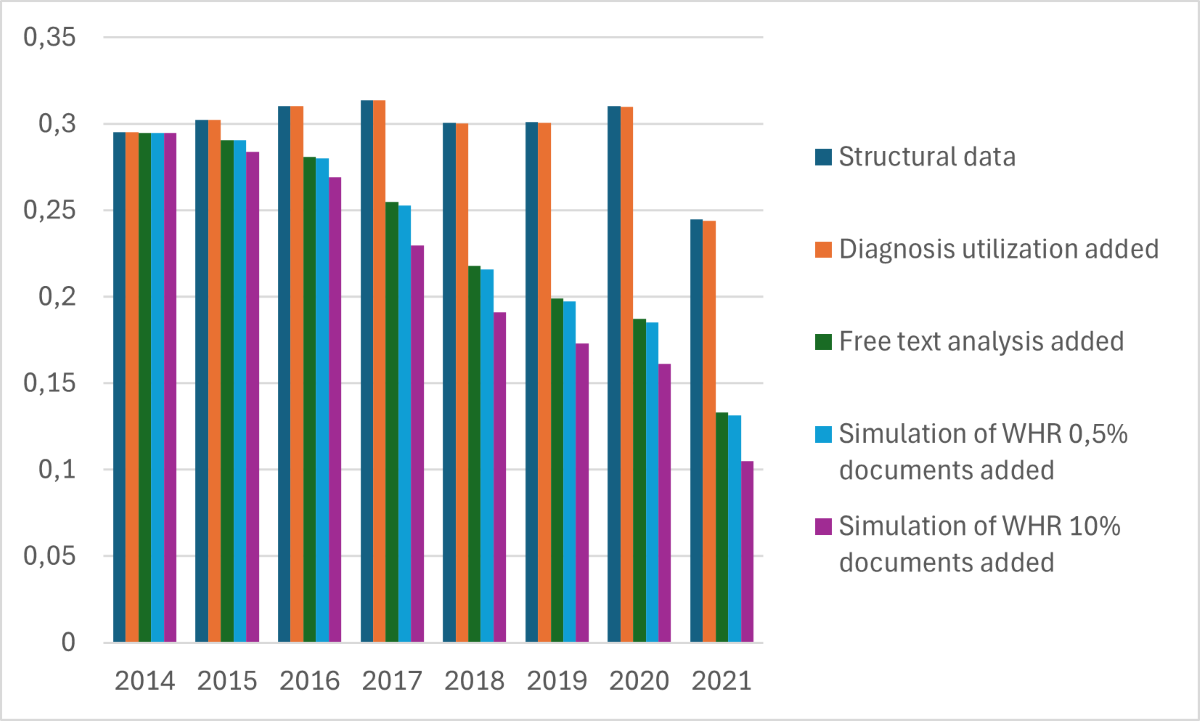
Average risk difference development between 2014 and 2021 with one year parameter lifetime. WHR: waist-hip ratio.

The average risk difference from our test runs is presented in Figures 5 and 6, with Figure 5 representing cases where an infinite timeline was used and Figure 6 representing cases where a 1-year lifetime was used. Risk difference is calculated by subtracting the minimum risk value from the maximum risk value. Smaller differences imply a more precise risk value. As we can see from the figures, the average risk difference has decreased as the data have been developed in Kanta. If we look at the case where structural data, diagnosis, and free text all have been used, the risk difference has been reduced by almost 20% points. We can also see that the free text use and the high availability simulation of WHR have had the largest impact on the risk difference.

In the test run, we calculated the most precise risk value for each person throughout their history in Kanta and categorized them by that risk value. Results can be seen in Multimedia Appendix 5.

As we can see, based on the results with only structural data, we can only categorize 341 out of 51,275 (<1%) individuals into any category. By adding diagnosis use for approximation, we did not achieve notable improvement in categorization. When adding the free-text analysis, we were able to categorize 21,020 out of 51,275 people (41%). Even though we did not manage to categorize any person into the high-risk category, we managed to identify individuals who were not at high risk. Due to the high availability of WHR information, we can categorize 38,279 out of 51,275 people (75%). A small number of individuals may be incorrectly placed in the high-risk category in the WHR simulation due to an inaccurate WHR value relative to their overall health status. However, this does not affect the overall categorization percentage, as it represents the proportion of individuals successfully categorized rather than the distribution across specific categories. At the original CLivD score research, fewer than 2% of individuals were identified as high-risk from a cohort of 25,760 individuals. Approximately 3% were identified as being at moderate risk in the original research. In our categorization, 2125 out of 51,275 individuals (4%) were classified into that category [8]. Based on these results, we can say that the results match because the CLivD score development dataset was from before 2012, while our dataset was from after 2014, and the risk for chronic liver disease has increased in the overall population in Finland.

### WHR Data

The WHR is among the most important variables for the CLivD risk score. Unfortunately, it cannot currently be obtained from Kanta data, as it is not supported in a structured format, and we were also unable to find it in the texts as well. As an alternative, we considered BMI, waist circumference, or hip circumference to predict the WHR. Of these, only BMI has meaningful availability in Kanta, so waist and hip circumferences were not considered further. BMI has been measured for 15,359 out of 51,275 (17%) individuals.

While associations between BMI and WHR can be found in the literature [16,17], the suitability of BMI as a predictor for severe liver disease has recently been disputed [18-21], with its suitability affected by gender and potentially other factors. Due to the seemingly complex and unclear nature of BMI and WHR interaction, we decided not to pursue WHR prediction for now.

For the analysis purpose, we simulated WHR data effect on the CLivD score risk model results in a few different scenarios. The first scenario involved obtaining the exact WHR data from the Kanta PDR, the second scenario involved the individual requesting their WHR data using the WHR groups, and the last scenario involved the individual measuring their exact WHR. These 3 scenarios were created because they serve as use case scenarios of the risk model differently.

The simulation was done by populating the timelines with WHR data. In the first case, 0.5% of all documents recorded in Kanta contained the WHR data, while in the second case, 10% of documents recorded in Kanta would have contained the WHR data. The first case represents the condition where WHR data would have the same kind of availability as all other basic physiological measurements currently in Kanta. The second case represents the condition where the WHR data would be highly available for individuals, for example, from other data sources. For the simulation, WHR values were generated using a normal distribution, with a mean of 0.96 for men, a mean of 0.84 for women, and a variance of 0.07. These values are found to be representative of the Finnish population based on research [22].

WHR as a measurement is slightly complicated for individuals to assess on their own, which raises the bar for using it in the CLivD score risk model. Because of this, we tested the cases to see if the simpler WHR categorization would give good results for risk categorization. The WHR categorization for a person could be easily implemented by displaying images of different body types and using these types to represent a WHR range. For the person, it would be easy to answer which image matches their own body. In this simulation, the exact WHR values were changed to the corresponding WHR category. After that, the average risk differences for the population were calculated for the cases, using 3 categories and 5 categories. WHR categorization definitions can be seen in Multimedia Appendix 3.

**Figure 7:**
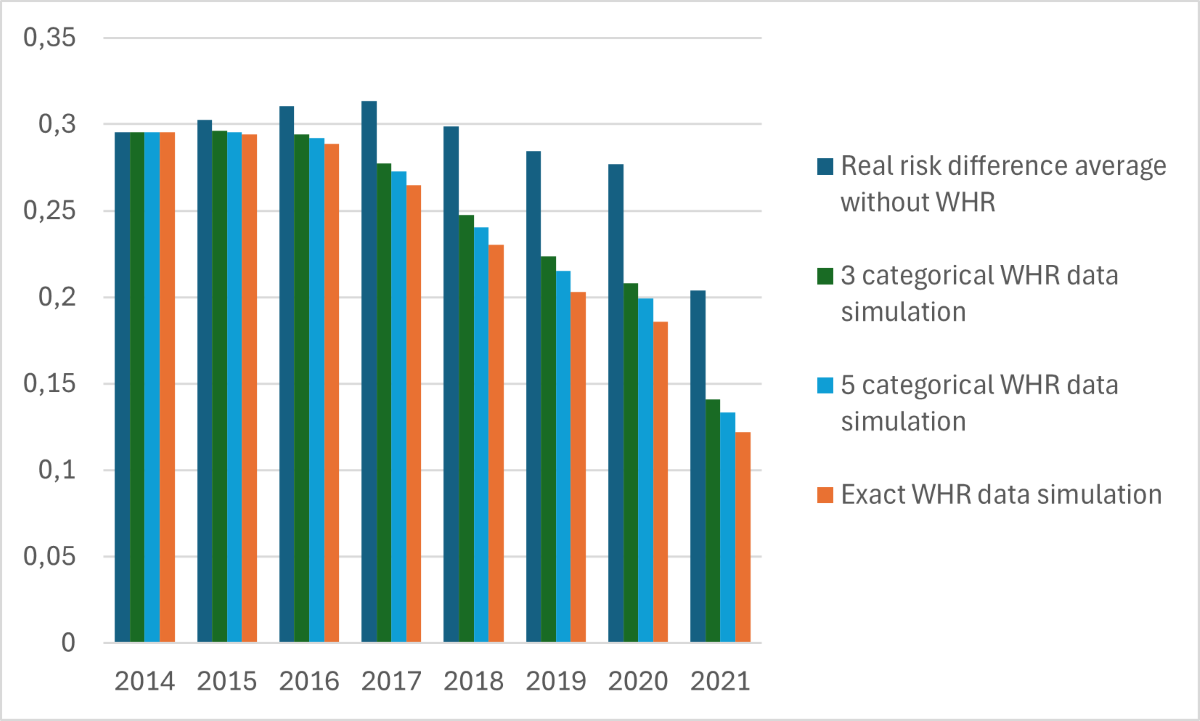
Waist-hip ratio (WHR) categorization impact to risk difference development between 2014 and 2021.

The results of the test run with the WHR simulation can be found in Figure 7. As we can see from the graph, there is no big change between the 3 categorical WHR data simulations and the exact WHR data simulation. If the WHR were requested by the individual, it would be simpler to assess body type using 3 categorical questionnaires rather than obtaining the exact WHR value.

## Discussion

### Principal Findings

We conclude that the current Kanta PDR data are not enough for precise risk calculation for the CLivD score or other risk models where obesity, smoking, and alcohol information are important risk factors. Our simulations show up to 14% improvement in risk detection when additional data sources are considered for obesity. Kanta shows excellent potential for implementing nationwide automated risk detection models that could result in improved disease prevention and public health.

Based on the results of this study, it is not possible to calculate precise risk scores using the CLivD risk model with the current data in Kanta PDR. However, risk categorization enables the possibility to use the CLivD risk model with Kanta PDR data so that it considers missing input parameters and enables future data development in Kanta. We noticed that risk categorization improved when the magnitude of input parameters was approximated using diagnosis information, and free text from the patient’s ongoing treatment report was used for input parameter parsing. When using the structured data from Kanta PDR as an input, we were able to identify only 33 out of 51,275 (<1%) individuals in the “low-risk” category and 308 out of 51,275 (<1%) individuals in “moderate-risk” category. When diagnosis use and free-text analysis were added to the model, we were able to identify 18,895 out of 51,275 (37%) individuals in the “low risk” category and 2125 out of 51,275 (4%) individuals in the “moderate risk” category. In both cases, we were not able to identify any individuals in the “high-risk” category, because of the missing WHR data. When we added the WHR simulation to the risk model, we started to identify “high-risk” individuals. We evaluated 3 scenarios to improve the coverage of WHR data in Kanta and these yielded the most substantial improvement in prediction accuracy.

### Comparison With Previous Work

In Finland, there is no systematic screening or automated risk assessment for chronic liver disease implemented in everyday health care. Diagnostics are quite random, and, in many cases, incidents are noticed during the treatment of other symptoms. Based on studies in Great Britain and Denmark, 70%-75% of cases of cirrhosis are only diagnosed when the patient ends up in the hospital’s emergency room with a serious complication of cirrhosis. In this case, the mortality rate is very high. Although similar research has not been conducted in Finland, the situation is presumed to be equally concerning.

There are automated methodologies implemented to diagnose liver fibrosis, but large-scale systematic analysis and screening are still yet to be implemented because of missing nationwide data. One randomized controlled study developed a care pathway for identifying advanced liver disease in patients with type 2 diabetes in Hong Kong and Malaysia. In that study, the results show that automation can lead to an increase in the number of referrals for patients with type 2 diabetes and abnormal fibrosis scores [23]. Another study showed that nationwide screening helps the early detection of liver cirrhosis [24]. Both of these studies have shown that screening and automated risk assessment can improve liver disease detection and it would be beneficial to implement nationwide health care analysis.

### Strengths and Limitations

The applied risk model used in this study has built-in flexibility for different availabilities of input data. This supports data quality development and enables flexible adaptation of different formats of input data. With this approach to risk assessment, we do not obtain the exact risk value from the model. However, this provides flexibility, as the risk categories offer valuable information as the exact risk value.

Approximation of smoking status and alcohol usage can be extracted using free text or various *ICD-10* or ICPC-2 diagnosis codes. These are still approximations and have the possibility for errors. In particular, it is unusual to have exact values from free text for alcohol usage, as it can be described using various words. As we were not able to use modern models for text analysis, such as generative large language models, we were still able to identify texts that refer to alcohol usage and smoking status. This means that although multiple texts refer to that information in free text, a consistent model is needed to analyze the data and achieve more reliable results. For that, the modern and more developed models would be a better option. To mitigate the analysis risk, we should target to convert alcohol usage free text into predefined categories, which would decrease the magnitude of errors in the analysis results.

Defining the new measurement type in the Kanta structural data does not require much work, but transferring the measurement results to Kanta can be time-consuming since the changes need to be implemented across all patient management systems that record actual data. In addition to changes to the actual systems to support the new measurements, changes in health care professional’s everyday patient care practices are also necessary to perform the actual measurement.

### Future Directions

To enhance the accuracy of risk model results, WHR data would be required. There are several possible ways to obtain WHR information, and their effectiveness depends on the use case. For example, Kanta PDR could have a new measurement type and WHR measurement could be added to general measurements in health care visits. It would take several years for Kanta to achieve broader population coverage since data are recorded only from individuals who use health care services. The other possible way would be to ask for information from the individual and use that in the risk model. There are a few ways to request a value from an individual; an exact value, a categorical question, or a camera measurement. The exact value asking is beneficial for the risk model because it will give the best results, but the measurement is quite difficult for individuals to perform on their own. Categorical questioning is easier to answer for the individual and can be implemented by showing photos of different body types, allowing the person to select the one that resembles them the most. This method would have the most errors, but it would be the easiest way for individuals. The last option would be to use a smartphone to measure WHR through the camera. The body can be identified using artificial intelligence, and the ratio can be calculated accurately based on an image. This requires a little bit of effort from the person but gives a precise risk value as a result.

As an alternative, we could show the WHR ranges assigning a patient to the low- or high-risk category, or we may consider developing a new risk function using BMI instead of WHR. As a potential future development, we may consider using Kanta PHR where patients themselves could record their WHR or ask categorized questions from the patient about the WHR.

Even though the risk model could not identify any high-risk patients with current Kanta data, it would still be important to implement this kind of risk model to use in everyday health care, because it would give real-time feedback about Kanta data quality development and guide health care professionals to make correct measurements for the patient. Patient management systems could inform health care professionals during patient visits about the exact measurements missing from the Kanta, preventing the calculation of the risk.

One option for finding missing data is using local data sources or mobile wellness apps. Both cases have their strengths and weaknesses. Local data sources for each patient management system may contain richer data than what is stored in Kanta, but integrating them into each local system requires a lot of work and maintenance. The National Electronic Health Record system in this case offers greater flexibility and simplifies data use. Changes should be made so that local data sources record all key values to Kanta. On the other hand, mobile wellness apps like Apple Health, Google Fit, and so on, store a lot of valuable data from personal health perspectives. The problem is that with the current legislation, nonmedical device data cannot be used in health care. This means that this offers possibilities only for personal health risk assessments and not for health care professional use cases.

In future work, the time dimension could be further used to track a patient’s risk over their whole patient history by plotting a graph of their minimum and maximum risk at each point in their timeline. This could help determine, for example, whether a change in their risk warrants an intervention or highlight a period of missing data in their history where the risk value is more uncertain.

In conclusion, the Finnish national electronic health record system has the potential to support automated risk detection models across the nation. However, capturing key chronic disease risk factors such as obesity, smoking, and alcohol use requires improvement.

## Appendix

Multimedia Appendix 1 Risk calculation formula.

Multimedia Appendix 2 Diagnosis mappings.

Multimedia Appendix 3 Category definitions.

Multimedia Appendix 4 Free text search keywords.

Multimedia Appendix 5 Risk categorization results.

## Abbreviations

CDA R2: Clinical Document Architecture Release 2
CLivD score: Chronic Liver Disease score
GGT: gamma glutamyl transferase
HL7: Health Level 7
ICD-10: International Statistical Classification of Diseases and Related Health Problems 10th Revision
ICPC-2: International Classification of Primary Care version 2
Kanta PDR: Kanta Patient Data Repository
RWD: real-world data
THL: Finnish Institute for Health and Welfare
WHR: waist-hip ratio

## Data Availability

Data were used under the secondary use of health care data law. The Finnish data permit authority Findata gave permission to use the data. The data were collected by Kela, the owner of Kanta Services. Data were processed in an audited secure environment with limited access. To have access to similar data, you must apply to Kanta Services data through Findata and define the collected data types. After the permission has been granted, the secure environment can be ordered, and the data will be delivered to it. The generative AI model ChatGPT was used only for clarifying the language.
