# Supplementary material for: Large-Scale Evaluation and Liver Disease Risk Prediction in Finland’s National Electronic Health Record System: Feasibility Study Using Real-World Data
Source: JMIR Med Inform. 2025 Apr 2;13:e62978. doi: 10.2196/62978 (PMC12004021; doi:10.2196/62978)
Supplement: Multimedia Appendix 1 [file medinform_v13i1e62978_app1.docx]

# Appendix 1: Risk calculation formula

${model}_{lab}$ and ${model}_{nonlab}$ are both described in detail at the publication [1] and we won’t repeat functions here. Here we describe the input data and functions application to real-world data used in this study. The first we define person input data model. Let’s denote input model to be $p$, where:

$$p_{Age}=\{p_{Age}\in\mathbb{R}\mid p_{Age}\geq40 \bigwedge p_{Age}\leq70\}$$

$$p_{Alcohol, min}=\left\{ \begin{aligned} p_{Alcohol}\in\mathbb{R}\mid p_{Alcohol}\geq0 \bigwedge p_{Alcohol}\leq50 \\ See Appendix 2 for alcohol diagnosis mappings \\ 0, if value not found and diagnosis not found \end{aligned} \right.$$

$$p_{Alcohol, max}=\left\{ \begin{aligned} p_{Alcohol}\in\mathbb{R}\mid p_{Alcohol}\geq0 \bigwedge p_{Alcohol}\leq50 \\ See Appendix 2 for alcohol diagnosis mappings \\ 50, if value not found and diagnosis not found \end{aligned} \right.$$

$$p_{GGT}=\{p_{GGT}\in\mathbb{R}\mid p_{GGT}\geq10 \bigwedge p_{GGT}\leq200\}$$

$$p_{WHR, min}=\left\{ \begin{aligned} p_{WHR}\in\mathbb{R}\mid p_{WHR}\geq0.7 \bigwedge p_{WHR}\leq1.2 \\ 0.7, if value not found \end{aligned} \right.$$

$$p_{WHR, max}=\left\{ \begin{aligned} p_{WHR}\in\mathbb{R}\mid p_{WHR}\geq0.7 \bigwedge p_{WHR}\leq1.2 \\ 1.2, if value not found \end{aligned} \right.$$

$$p_{Sex}=\left\{ \begin{aligned} Female \\ Male \end{aligned} \right.$$

$$p_{Diabetes, min}=\left\{ \begin{aligned} 0, if diagnosis or medication is not found or fasting glucose<0.7mmol/l \\ 1, if diagnosis or medication is found or fasting glucose\geq0.7mmol/l \\ 0, ifdiagnosis, medication or fasting glucose measurement not found \end{aligned} \right.$$

$$p_{Diabetes, max}=\left\{ \begin{aligned} 0, if diagnosis or medication is not found or fasting glucose<0.7mmol/l \\ 1, if diagnosis or medication is found or fasting glucose\geq0.7mmol/l \\ 1, ifdiagnosis, medication or fasting glucose measurement not found \end{aligned} \right.$$

See the original paper [1] for complete diabetes definitions.

$$p_{Smoking, min}=\left\{ \begin{aligned} 0, if person is non smoker \\ 1,if person is smoker \\ 0, if smoking status is not found \end{aligned} \right.$$

$$p_{Smoking, max}=\left\{ \begin{aligned} 0, if person is non smoker \\ 1,if person is smoker \\ 1, if smoking status is not found \end{aligned} \right.$$

For the complete smoking information data transformation from diagnosis information see the Appendix 1 and 2.

The next we define functions for calculating the minimum and maximum risk as follows:

$${CLivD}_{Min}=\left\{ \begin{aligned} {model}_{lab}\left( p_{min} \right), ifp_{GGT}\in[10-200] \\ {model}_{nonlab}(p_{min)} \end{aligned} \right.$$

$${CLivD}_{Max}=\left\{ \begin{aligned} {model}_{lab}\left( p_{max} \right), ifp_{GGT}\in[10-200] \\ {model}_{nonlab}(p_{max)} \end{aligned} \right.$$

Risk category is defined as follows:

$$CLivD\left( p \right)=\left\{ \begin{aligned} Low risk, {CLivD}_{Min}\left( p_{min} \right)\leq5\% \bigwedge{CLivD}_{Max}\left( p_{max} \right)\leq5\% \\ Moderate risk, {5\%<CLivD}_{Min}\left( p_{min} \right)<10\% \bigwedge{CLivD}_{Max}\left( p_{max} \right)>5\% \\ High-risk, {CLivD}_{Min}\left( p_{min} \right)\geq10\% \bigwedge{CLivD}_{Max}\left( p_{max} \right)\geq10\% \\ Not Specified, {CLivD}_{Min}\left( p_{min} \right)>5\% \bigwedge{CLivD}_{Max}\left( p_{max} \right)\geq10\% \end{aligned} \right.$$

## References

[1] Development and validation of a model to predict incident chronic liver disease in the general population: The CLivD score, Åberg, Fredrik et al., Journal of Hepatology, Volume 77, Issue 2, 302 - 311
