# Supplementary material for: Large-Scale Evaluation and Liver Disease Risk Prediction in Finland’s National Electronic Health Record System: Feasibility Study Using Real-World Data
Source: JMIR Med Inform. 2025 Apr 2;13:e62978. doi: 10.2196/62978 (PMC12004021; doi:10.2196/62978)
Supplement: Multimedia Appendix 2 [file medinform_v13i1e62978_app2.docx]

# Appendix 2 Diagnosis mappings

| **Input parameter type** | **Diagnosis code system** | **Diagnosis code** | **Gender** | **Minimum value** | **Maximum value** |
| --- | --- | --- | --- | --- | --- |
| Alcohol usage | ICD-10 | F10* | Male | 23 | 49 |
|  |  |  | Female | 12 | 49 |
|  | ICPC-2 | P15 | Male | 23 | 49 |
|  |  |  | Female | 12 | 49 |
|  | ICPC-2 | P16 | Male | 23 | 49 |
|  |  |  | Female | 12 | 49 |
| Smoking information | ICD-10 | Z72.0 | Both | true | true |
|  | ICPC-2 | P17 | Both | true | true |
