# Supplementary material for: Large-Scale Evaluation and Liver Disease Risk Prediction in Finland’s National Electronic Health Record System: Feasibility Study Using Real-World Data
Source: JMIR Med Inform. 2025 Apr 2;13:e62978. doi: 10.2196/62978 (PMC12004021; doi:10.2196/62978)
Supplement: Multimedia Appendix 3 [file medinform_v13i1e62978_app3.docx]

# Appendix 3: Input parameter categories used in Chronic Liver Disease score calculation

**Table 1.** Alcohol usage categories used in Chronic Liver Disease score calculation

| Description (category index) | | Min alcohol usage in servings | Max alcohol usage in servings |
| --- | --- | --- | --- |
| **Abstainer (0)** | | | |
|  | Male | 0 | 0 |
|  | Female | 0 | 0 |
| **Light to moderate use (1)** | | | |
|  | Male | 1 | 14 |
|  | Female | 1 | 7 |
| **Moderate or Heavy use (2)** | | | |
|  | Male | 14 | 49 |
|  | Female | 7 | 49 |
| **Risk user (3)** | | | |
|  | Male | 23 | 49 |
|  | Female | 12 | 49 |

**Table 2.** Waist-Hip ratio categories used in Chronic Liver Disease score risk calculation.

| Waist-hip ratio category | 3 categories, Waist-hip ratio range | 5 categories, Waist-hip ratio range |
| --- | --- | --- |
| 1 | <0.9 | <0.82 |
| 2 |  | 0.82-0.94 |
| 3 | 0.9-1.1 | 0.95-1.06 |
| 4 | >1.1 | 1.07-1.18 |
| 5 |  | >1.19 |
