# Supplementary material for: Large-Scale Evaluation and Liver Disease Risk Prediction in Finland’s National Electronic Health Record System: Feasibility Study Using Real-World Data
Source: JMIR Med Inform. 2025 Apr 2;13:e62978. doi: 10.2196/62978 (PMC12004021; doi:10.2196/62978)
Supplement: Multimedia Appendix 4 [file medinform_v13i1e62978_app4.docx]

# Appendix 4: Free text search keywords

| **Parameter to be found** | **Text search Regex** | **Paraemeter value if text found** |
| --- | --- | --- |
| Smoking | koskaan tupak | false |
|  | ei (?:ole\|polt\w*)?\s*tupak | false |
|  | lopetta\w*\s* tupak | true |
|  | lopett\w*\s*tupak | false |
|  | tupak\S*\s*(?:lopettaminen\|lopettamis\w*) | true |
|  | tupak\S*\s*(?:on\|polt\w*)?\s*lopett | false |
|  | tupakoimaton | false |
|  | tupakoiva | true |
|  | (?:aiemmin\|ennen\|aikaisemmin\|nuore\w*\|ex-?)\s*tupak | false |
|  | tupakoi[,\.] | true |
|  | tupak\S*\s*(-\|ei) | false |
|  | tupak\S*\s*(\+\|kyllä) | true |
|  | (?:savuk\|tupak)\S*:?\s?(\d+) | true |
|  | tupak\w*\W*(?:epäsään\|sään\|joskus\|toisinaan\|  ajoittain\|satunnaisesti\|silloin tällöin\|päivittäin) | true |
|  | tupakoi \d+ (savu\|kpl) | true |
|  | tupakoitko päivittäin\?:?\s?kyllä | true |
| Number of alcohol servings | käytä alkohol | 0 (Appendix 1 category) |
|  | ei\s*\w*\s*alkohol | 0 (Appendix 1 category) |
|  | alkohol\S*\s*(-\|ei) | 0 (Appendix 1 category) |
|  | alkoh\S*\s*(?:juo\S*\|käyt\S*)?\s*(?:on\|:\|ollut)?\s*  (?:hyvin\|erittäin\|todella\|vain\|tosi)?\s*(?:kohtuu\|vähä  \|toisinaan\|joskus\|satunn\|harv\|juuri\|maltill\|lopett) | 1 (Appendix 1 category) |
|  | alkoh\S*\s*(?:juo\S*\|käyt\S*)?\s*(?:on\|:\|ollut)?\s*  (?:hyvin\|erittäin\|todella)?\s*(?:runs\|palj\|reilu\|päiv) | 3 (Appendix 1 category) |
|  | alkohol\S*\s*(?:käytön)?\s*(?:ongelma\|liika\|haitall  \|väär\|riippu\|laukaisema\|aiheuttama\|seurauk) | 3 (Appendix 1 category) |
|  | vieroitus\|vierotus\|päihtymys\|putki\|katkaisu | 3 (Appendix 1 category) |
|  | pitkäaik\S*\s*alkohol | 3 (Appendix 1 category) |
|  | runsa\w*\s*alkohol | 3 (Appendix 1 category) |
|  | audit(?:\s*indeksi\s*)?:?\s?(\d+) | Results match to Appendix 1 category  Audit Score 0 -> 0  Male and 0 < Audit Score < 8 -> 1  Female and 0 < Audit Score < 10 -> 1  Male and 8 <= Audit Score <= 10 -> 2  Female and 6 <= Audit Score <= 10 -> 2  Audit Score >= 11 -> 3 |
|  | (\d+)\s*annos\w*\s?/?\s?(\S*) | Servings converted to weekly servings and result matched to correct Appendix 1 category. |
