# Supplementary material for: Large-Scale Evaluation and Liver Disease Risk Prediction in Finland’s National Electronic Health Record System: Feasibility Study Using Real-World Data
Source: JMIR Med Inform. 2025 Apr 2;13:e62978. doi: 10.2196/62978 (PMC12004021; doi:10.2196/62978)
Supplement: Multimedia Appendix 5 [file medinform_v13i1e62978_app5.docx]

# Appendix 5: Risk categorization results

**Table 1.** Risk categorization results in an infinite life cycle (N=51,275).

| Risk category | Structural data | Diagnosis use added | Free text analysis added | Simulation of Waist-Hip -ratio 0,5% documents added | Simulation of Waist-Hip -ratio 10% documents added |
| --- | --- | --- | --- | --- | --- |
| Low risk | 33 | 33 | 18,895 | 19,341 | 33,760 |
| Moderate risk | 308 | 310 | 2125 | 2351 | 4495 |
| High-risk | 0 | 0 | 0 | 6 | 24 |
| Not specified | 50,934 | 50,932 | 30,255 | 29,577 | 22,420 |
